# Intramedullary Schwannoma in the Cervical Region: A Case Report

**DOI:** 10.7759/cureus.74632

**Published:** 2024-11-27

**Authors:** Sukru Oral, Ali Sahin, Ninetullah A Durmus, Halil Ulutabanca, Kemal Koç

**Affiliations:** 1 Neurosurgery, Erciyes University Faculty of Medicine, Kayseri, TUR

**Keywords:** intradural extramedullary mass, schwann cell neoplasm, spinal cord tumor surgery, spine microsurgery, spine oncology

## Abstract

Intramedullary schwannomas are a type of benign spinal cord tumor that originates from the Schwann cells of the nerve sheath. They are relatively rare and typically occur within the spinal cord itself, rather than in the surrounding tissue. Treatment options for cervical intramedullary schwannomas include surgical removal of the tumor, radiation therapy, and observation. Surgery is often considered the first-line treatment, as complete removal of the tumor can provide the best outcome for patients. However, the choice of treatment will depend on various factors such as the size and location of the tumor. In this study, a case of intramedullary schwannoma located in the cervical region is presented and the literature is reviewed. The literature review searched through some databases, including PubMed, Elsevier, and Ovid Medline, and found a total of 83 cases of intramedullary cervical schwannomas reported in 70 articles since 1932. Forty-nine of the cases were male, 34 of them were female and the mean age was 38.9 years. The cases associated with neurofibromatosis were not included in this study. Astrocytoma, ependymoma, and schwannoma are all tumors that can occur within the spinal cord, but they have different origins, different characteristics, and different growth patterns. Astrocytomas and ependymomas are more commonly found in intramedullary locations within the spinal cord, compared to schwannomas. In summary, when intramedullary tumors are suspected, it's important to consider schwannomas as a differential diagnosis.

## Introduction

Astrocytomas and ependymomas constitute 80% of intramedullary spinal tumors. The third frequent is hemangioblastomas. Although schwannomas constitute approximately 30% of primary spinal tumors, the intramedullary form is much rarer [1,2] because the Schwann cell from which the tumor originates is not found in the intramedullary area and brain parenchyma. While spinal schwannomas are rarely located in the thoracic and lumbar regions, they are more common in the cervical region [2]. In addition, intramedullary schwannomas without neurofibromatosis have been reported even more rarely [3,4]. The purpose of the study is to provide information on the diagnosis and management of this rare condition, as well as to add to the existing knowledge on intramedullary tumors in the cervical spinal region. Intramedullary tumors located in the cervical spinal region can be challenging to remove surgically due to the proximity of important neural and vascular structures. Therefore, the surgical approach and technique used for this patient may have been unique and of particular interest to the surgeons.

## Case presentation

Case report

**Figure 1:**
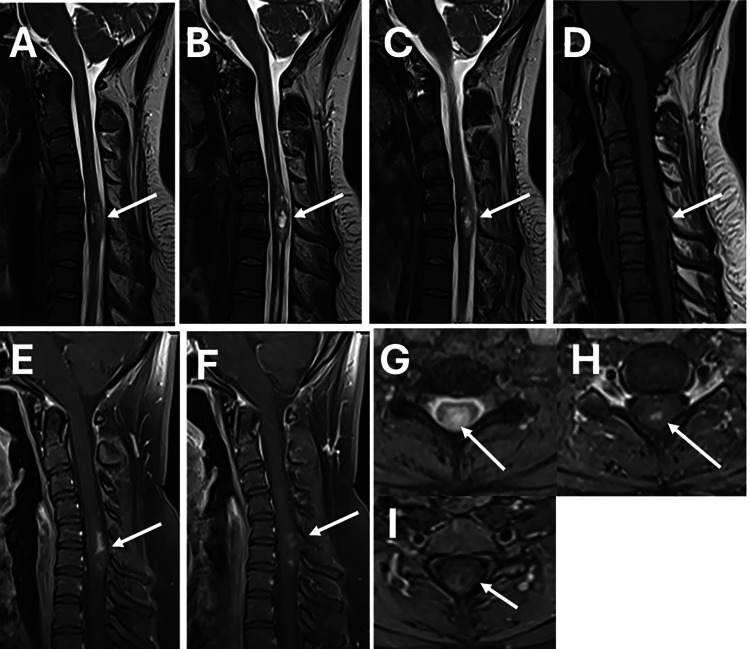
Preoperative magnetic resonance images of the patient (A) The lesion enlarging the spinal cord at the C5-6 level should be seen on T2-weighted MRI (white arrow) (B) On another T2-weighted MR slice at the same level, the lesion is heterogeneous (iso and hyperintense) (white arrow) (C) On another T2-weighted MR slice at the same level (white arrow) (D) On T1-weighted MR image, the center of the mass is hypointense and the periphery is isointense (white arrow) (E,F) T1 contrast-enhanced MR images show that the mass is irregularly circumscribed and heterogeneously contrasted (white arrow) (G) T2 axial MR image shows that the lesion center is hyperintense and intramedullary (white arrow) (H,I) T1 axial contrast-enhanced MR images show heterogeneous contrast enhancement and intramedullary localization of the spinal cord (white arrow)

A 37-year-old female patient was admitted to our clinic with complaints of neck, arm, and shoulder pain and numbness in the thumb for about five months. The patient's complaints were more on the right side. On the physical and neurological examination of the patient, hypoesthesia in bilateral C5-6 dermatomes, increased deep tendon reflexes, and positive Hoffmann reflex were found. Apart from these, no additional features were detected in routine laboratory tests. Cervical radiography and computed tomography were evaluated as normal. In the magnetic resonance imaging (MRI) of the patient's cervical region, the image of an intramedullary localized mass at the C5-6 level, involving the posterior and central spinal cord, with irregular borders, shows that the solid component is isointense in T1-weighted sagittal sections, the cystic component is hypointense, and in contrast-enhanced T1-weighted images the solid component is isointense and the cystic component circumferential contrast. On T2-weighted examination, the cystic part of the cystic intradural mass was evaluated as hyperintense and the solid part as isointense (Figure 1). The patient was pre-diagnosed with an intramedullary mass, possibly ependymoma, the necessary preparations were made, and she was taken into operation.

Surgical technique

**Figure 2:**
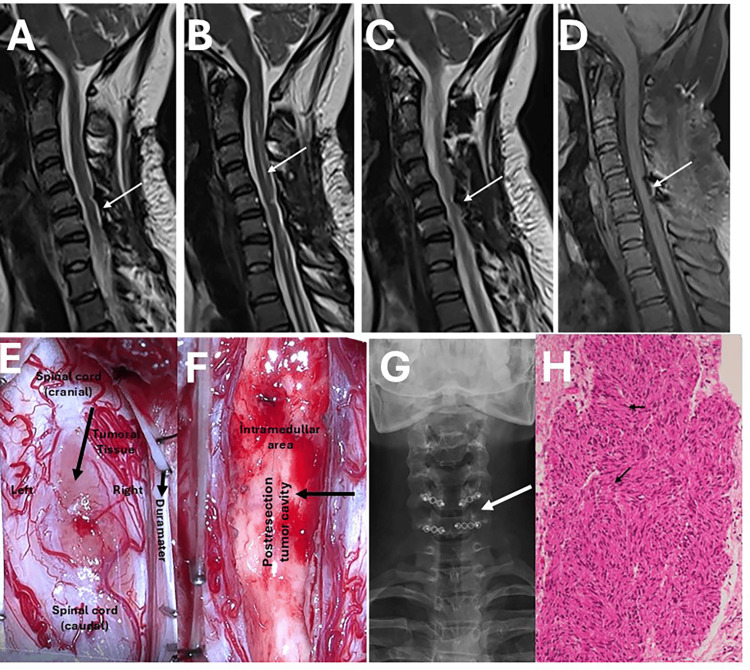
Postoperative magnetic resonance images, direct radiographs, intraoperative surgical photographs and pathologic imaging (A,B,C) In the T2-weighted MRI sections taken in the third month postoperatively, it is observed that the mass is totally removed (white arrow) (D) T1 contrast-enhanced MR image does not show any contrast enhancing lesion (white arrow) (E) After opening the duramater from the midline on both sides, the spinal cord and the tumor tissue located in it can be seen (black arrow) (F) After tumor removal, a tumor cavity is observed in the intramedullary area (black arrow) (G) C5 and C6 laminoplasty appearance on cervical X-ray (white arrow) (H) Histopathological appearance of tumor tissue. In the center of the picture (black arrows) there are areas of dense spindle cells (Antoni A) arranged in the form of fascicles and whorl structures, sometimes showing palisadic arrangement, and more peripheral areas of cell-poor loose connective tissue (Antoni B). S-100 is diffusely positive in neoplastic cells (immunohistochemical staining).

The patient was operated in the prone position under general anesthesia. After neuromonitoring, the level of C5-6 was marked with C-Arm Scope. A dorsal midline skin incision was made, and the subcutaneous tissue and fascia were opened. The paravertebral muscles were stripped from the bilateral subperiosteal bone structure. C5 and C6 laminotomy were performed. After removing the lamina and flavum, the dura was opened with a linear incision. And the mass was reached. The mass was pink greyish in colour, hard consistency, had a cleavage plan around it (Figure 2), and the tumor tissue could hardly be separated from the neural tissue. The tumor was slightly to the right of the midline. The tumor center was shrunk by debulking with an ultrasonic aspirator, and the tumor was dissected from the neural tissue with a microsurgical technique and the tumor was totally excised. After haemostasis, the dura mater primary was closed. C5-6 laminoplasty was performed using a miniplate and screw. The opened folds were closed primarily, respectively, and the surgery was terminated.

In the pathological evaluation, the mass was determined as schwannoma. In the postoperative follow-up, the clinical findings of the patient were completely improved. No residual or recurrent mass was found in the control cervical MRI performed in the third month after the operation (Figure 2).

## Discussion

Schwannomas constitute the majority of intradural nerve sheath tumors. Schwannomas may occur sporadically or as a component of hereditary diseases. Because they are benign, they tend to grow slowly [3].

There is no definite information about how intramedullary schwannoma occurs. However, there are hypotheses such as central displacement of Schwann cells during embryological development, a proliferation of Schwann cells along intramedullary perivascular nerve plexuses or along aberrant nerve fibers, neoplastic developments at the point where dorsal cocci lose their sheath at the entrance to the piamater, and transformation of tumoral pia cells in neoplastic schwann cells [5]. In our case, we think that the schwannoma originated from the dorsal roots because it was located posterior to the spinal cord in surgical images and MRI sections. In the literature, we observed that in some cases reported, the symptoms were more dominant on the side of the tumor [6,7].

They tend to grow slowly and have a relatively low rate of recurrence after surgical removal. They tend to be closely related to a specific nerve root, unlike the other tumors and can cause compression of the nerve root and related symptoms. Therefore, when they reach large sizes, they give clinical findings. The clinical findings of intramedullary tumors in the spinal cord can vary depending on the level of the spinal cord that is affected. Tumors located in the cervical spinal cord, for example, can cause symptoms such as neck pain, weakness or numbness in the arms or legs, and difficulty walking or coordination. Tumors located in the thoracic or lumbar regions of the spinal cord, on the other hand, can cause symptoms such as back pain, weakness or numbness in the legs, and difficulty walking or coordination. Patients generally apply to the hospital with complaints of pain and sensory defects [8-10]. Similarly, studies have shown that intramedullary schwannomas are more common in men and in the fourth decade of life. If the mass reaches very large sizes and compresses the spinal cord, myelopathy findings may develop. Although pain and sensory defect were prominent in our patient, increased deep tendon reflexes and Hoffman reflexes were evaluated as examination findings in favour of myelopathy. In addition, the symptoms were more dominant on the right side. In MRI sections and surgical images, the tumor tissue caused more compression on the right side of the spinal cord. Therefore, when we evaluate it under the current findings, we think that the tumor does not originate from the central canal in the midline but originates from the dorsal roots [11].

Contrast-enhanced magnetic resonance imaging is the most sensitive and specific radiological imaging method in these tumors. These tumors often show homogeneous enhancement. In addition, heterogeneous staining, intratumorally cyst, haemorrhage, or necrosis can also be seen. They are mostly located in the cervical region. It is very difficult to diagnose intramedullary schwannomas radiologically and to make a differential diagnosis from other tumors. It can be seen as isointense or hypointense on T1-weighted images, while hyperintense on T2-weighted images. In contrast-enhanced MRI, the tumor border maintains sharp, regular, and homogeneous contrast. Thanks to the contrast, the tumor can be distinguished from the surrounding cystic structure and oedema [12].

The MRI features of our case are compatible with the literature in terms of heterogeneous contrast enhancement and intratumorally cysts [3,13]. But it differs from others in having irregular borders.

Intramedullary ependymomas generally enlarge symmetrically within the spinal cord and are centrally located. On T1-weighted MRI scans, ependymomas may be hypointense or isointense in the spinal cord and hyperintense on T2-weighted MRI sections. The development of ependymomas is usually intense and their borders are sharply demarcated. Astrocytomas, on the other hand, are more irregularly circumscribed and have heterogeneous contrast enhancement. Astrocytomas are most common in the thoracic region, second most equently in the cervical region, and schwannomas are located more frequently in the cervical region. Surgical resection is more difficult than other tumors because they are irregularly circumscribed [14-17]. In the surgery of cervical intradural intramedullary tumors, posterior approaches are generally preferred, and very rarely anterior approaches. While laminectomy was generally preferred in posterior surgical approaches before 2000, techniques such as laminoplasty, hemilaminectomy, and hemi-laminoplasty were used more widely in the following years [3,12,18]. Similarly, neuromonitoring has been used routinely in intramedullary tumor surgery in recent years. Until today, detailed information about the surgical technique has not been given in the studies on the subject, and it has been mentioned that laminectomy is used in most studies. Considering the long-term complications after laminectomy, especially in young patients, the choice of laminoplasty or hemilaminectomy has become widespread in such cases.

Our case was operated via posterior surgical intervention, intraoperative neuromonitoring, and laminoplasty. No complications developed during or after the operation.

Since intramedullary schwannomas are very rare tumors, a limited number of cases have been reported in the literature. In our literature review, approximately 180 cases of intradural intramedullary schwannoma have been reported since 1932, and it has been shown that approximately 83 of them are in the cervical region.

## Conclusions

Schwannomas should be considered in the differential diagnosis when evaluating intradural and intramedullary tumors. In addition, schwannoma should be kept in mind, especially in cases where the symptoms are evaluated as more dominant on the right or left side. 
